# Evaluating the Relationship Between Programmed Death Ligand-1 (Clone: 22C3), Anaplastic Lymphoma Kinase (D5F3), and C-ros Oncogene 1 (OT11A1) Expression in Lung Adenocarcinoma

**DOI:** 10.7759/cureus.101737

**Published:** 2026-01-17

**Authors:** Kartavya K Verma, Amit Bugalia, Ajoy K Behera, Nighat Hussain

**Affiliations:** 1 Department of Pathology, Shri Rawatpura Sarkar Institute of Medical Sciences and Research, Raipur, IND; 2 Department of Pathology and Laboratory Medicine, All India Institute of Medical Sciences, Raipur, IND; 3 Department of Pulmonary Medicine and Tuberculosis, All India Institute of Medical Sciences, Raipur, IND

**Keywords:** anaplastic lymphoma kinase (alk), c-ros oncogene 1 (ros1), lung adenocarcinoma (adc), molecular oncology, programmed death ligand 1 (pd-l1)

## Abstract

Objective

This study investigates the relationship between programmed death ligand-1 (PD-L1), anaplastic lymphoma kinase (ALK), and c-ros oncogene 1 (ROS1) expression in lung adenocarcinoma (ADC).

Methods

Sixty-two cases were analyzed for ALK, ROS1, and PD-L1 expression using immunohistochemistry. Statistical evaluations were performed using chi-square and Fisher’s exact tests.

Results

Among the 62 cases, 24 (39%) were identified as PD-L1 positive, while four (6.45%) and three (4.84%) demonstrated ALK and ROS1 positivity, respectively, highlighting the molecular heterogeneity of lung adenocarcinoma. A significant association was observed between PD-L1 expression and the clinical stage of the disease (p = 0.048, df = 5, *χ²* = 11.12), suggesting the potential of PD-L1 as a biomarker for tumor progression. Conversely, no significant associations were found between clinical stage and ROS1 (p = 0.61, df = 5, χ² = 3.62) or ALK positivity (p = 0.13, df = 5, χ² = 8.58), indicating limited relevance of these genetic alterations to disease staging. Correlations between PD-L1 positivity and ALK or ROS1 positivity were also not definitive; the odds ratios were approximately 33.1 (95% confidence interval (CI): 2.1-518.5) for ALK (p ≈ 0.058) and 27.1 (95% CI: 1.7-424) for ROS1 (p ≈ 0.058). Only three cases exhibited copositivity for both ALK and ROS1, with an odds ratio of about 1.17 (95% CI: 0.20-6.58) and a p-value of approximately 0.95, indicating no significant association.

Conclusion

The expression of PD-L1 is independent of ALK and ROS1 alterations in lung adenocarcinoma, suggesting that these biomarkers do not significantly correlate with one another.

## Introduction

Lung carcinoma, a malignant epithelial tumor originating from bronchial and alveolar cells, begins with genetic alterations such as oncogene activation or tumor suppressor inactivation. Notably, adenocarcinomas derive from proliferating type II pneumocytes and often evolve from precursor lesions such as adenoma or adenocarcinoma (ADC) in situ, as defined by World Health Organization (WHO) criteria. Emerging molecular insights emphasize that complex genetic and environmental interactions drive its development, highlighting the importance of early molecular detection for targeted therapies. The correlation among these mutations has been explored only minimally in the Indian population.

The echinoderm microtubule-associated protein-like 4-anaplastic lymphoma kinase (EML4-ALK) gene fusion, common in lung adenocarcinoma, responds well to targeted tyrosine kinase inhibitors (TKI). Notably, this alteration is more prevalent among smokers than epidermal growth factor receptor (EGFR) mutations. Detection primarily employs fluorescence in situ hybridization (FISH) and immunohistochemistry (IHC) with the Food and Drug Administration (FDA)-approved ALK D5F3 clone. A positive IHC result with this clone typically eliminates the need for confirmatory FISH testing, streamlining the diagnostic process and enabling timely, personalized therapy [1]. For tumors harboring ALK rearrangements, ALK inhibitors are the preferred first-line therapy. Recent phase III studies comparing the first-generation ALK TKI, crizotinib, to standard chemotherapy in treatment-naive patients demonstrated notable improvements in progression-free survival, higher response rates, and better overall quality of life, establishing ALK inhibitors as a superior initial treatment option [2].

The c-ros oncogene 1 (ROS1) is another tyrosine kinase that can be rearranged in non-small cell lung cancer (NSCLC), often involving various fusion partners. In lung adenocarcinoma, ROS1 mutations are frequently linked to a history of smoking and show a good response to targeted oral tyrosine kinase inhibitors. Detection methods include FISH and IHC; however, IHC has lower sensitivity, making FISH the preferred confirmatory test. Currently, there are no FDA-approved IHC assays specifically for ROS1 mutation detection in NSCLC [1]. The ROS1 tyrosine kinase is highly responsive to targeted therapies such as crizotinib, which inhibits ROS1 and MET, and entrectinib, a dual inhibitor of ROS1 and tropomyosin receptor kinases (TRK). Both drugs have received FDA approval for treating patients with ROS1 gene rearrangements, demonstrating their effectiveness in managing this specific molecular subtype of lung cancer [3].

Programmed death ligand-1 (PD-L1) is an immunoregulatory protein expressed on tumor cells that helps suppress T-cell activity, allowing the cancer to evade immune attack. The level of PD-L1 expression is typically interpreted based on tumor cell positivity, with over 50% considered high expression and less than 1% regarded as negative. This evaluation is essential for guiding treatment decisions, as the FDA has approved the use of immunohistochemistry for CD274 to accurately assess PD-L1 status in tumors [1]. Standard evaluation of PD-L1 expression is advised for all newly diagnosed cases of advanced NSCLC, as it plays a crucial role in selecting optimal immunotherapy options. Patients with tumor PD-L1 expression levels of 50% or more are advised to undergo treatment with single-agent pembrolizumab or atezolizumab. Clinical studies have shown that these therapies notably improve overall survival (OS) rates compared to standard chemotherapy in this specific patient group [4].

Therapies targeting specific genetic mutations have revolutionized lung cancer treatment, especially in inoperable cases. Key driver mutations such as EGFR or HER1, ALK, and ROS1 are critical for personalized management, with emerging markers such as PD-L1 and HER2neu also gaining importance. While FDA-approved drugs are frontline options, genetic testing via next-generation sequencing (NGS), FISH, polymerase chain reaction (PCR), or IHC is essential. In regions with limited resources, clone-specific IHC offers a practical screening alternative, enabling broader access to precision medicine despite infrastructural challenges. Performing molecular testing in every case can be challenging due to limitations in available tissue and the high costs associated with testing. The College of American Pathologists (CAP) has recommended specific testing methods and sequencing for these molecular analyses. Once a diagnosis of NSCLC adenocarcinoma is established, testing for mutations such as EGFR, ALK, PD-L1, and ROS1 is advised. This study found that the correlation with these targeted mutations in ADC offers therapeutic benefits [5]. The study is designed to explore potential relationships among PD-L1, ALK, and ROS1 positivity in ADC. If a correlation between PD-L1 and any of these alterations is identified, particularly in the context of mutations in India, it could facilitate more timely decisions regarding treatment protocols for targeted therapies.

## Materials and methods

Patient

This study included diagnosed primary lung adenocarcinoma cases confirmed by histopathology and IHC in patients over 18 years of age, received at the Department of Pathology and Laboratory Medicine, All India Institute of Medical Sciences (AIIMS), Raipur, between 2019 and 2021. Cases of squamous cell carcinoma (SCC), small cell carcinoma, or other malignancies were excluded.

IHC procedure

The process begins by cutting tissue sections onto poly-L-lysine-coated slides. The slides are then placed on a warming plate at 58°C-60°C for 10 minutes to ensure proper adhesion. The sections are subsequently dewaxed by immersing the slides serially in xylene for 10 minutes twice, followed by two-minute washes in 100%, 90%, and 70% alcohol solutions, with a two-minute water wash afterward. Antigen retrieval is performed by heating the slides in Tris-EDTA buffer (pH 8.0) using a microwave, first at 600W for five minutes, then at 450W for five minutes, and again at 450W for five minutes, checking buffer levels regularly. The slides are then rinsed under running tap water until they reach the approximate room temperature. To block endogenous peroxidase activity, the sections are incubated with 3% hydrogen peroxide for six minutes, followed by a five-minute wash in Tris buffer saline (pH 7.2-7.4). Next, the primary antibody is applied and incubated for 30-60 minutes. After this, the slides are washed in Tris buffer saline for five minutes, then incubated with an Envision (enzyme-linked polymer) labeled polymer for 30 minutes, followed by another five-minute wash. The slides undergo two additional five-minute washes in buffer, after which chromogen (3,3'-diaminobenzidine (DAB)) is applied for eight minutes to visualize the antigen. Following chromogen development, the slides are washed again in buffer and then in distilled water for five minutes each. The sections are counterstained with hematoxylin for five minutes, then dehydrated with two changes of 100% isopropanol alcohol for two minutes each, cleared in xylene, and finally mounted with dibutylphthalate polystyrene xylene (DPX) before labeling for analysis.

IHC study

Positive ALK expression was identified by strong granular staining in the cytoplasm of tumor cells, regardless of the percentage. ALK (clone: D5F3, rabbit monoclonal; Dako, Carpinteria, CA), with ALK-positive anaplastic large cell lymphoma as the validation control, was confirmed via PCR. Positive ROS1 expression was identified by strong granular staining in tumor cell cytoplasm. ROS1 (clone: OT11A1, rabbit monoclonal, Thermo, CA), with reactive pneumocytes and ROS1-positive tumor cells serving as controls, were also validated by PCR. PD-L1 expression levels were quantified using the Tumor Proportion Score (TPS): TPS < 1% indicated no expression, 1% ≤ TPS < 50% indicated low expression, and TPS ≥ 50% indicated high expression [6]. PD-L1 (clone: 22C3, rabbit monoclonal, Thermo), with tonsillar crypt epithelium as the internal control.

Data analysis

Data were analyzed using SPSS version 23.0 (IBM Corp., Armonk, NY). Fisher’s exact and chi-square tests were applied to assess the significance of observed associations, ensuring robust statistical validation.

## Results

In this study of 62 lung ADC cases, 46 (74.19%) were men and 16 (25.81%) were women. PD-L1 positivity was observed in 24 (38.7%) cases, with a significant association across clinical stages (χ² = 11.12, p ≈ 0.048, df = 5), indicating that PD-L1 expression varies notably with disease progression. Conversely, ALK positivity was found in four (11.3%) cases and showed no significant correlation with stage (χ² = 8.58, p ≈ 0.13, df = 5). Similarly, ROS1 positivity was detected in three (9.7%) cases with no significant association with stage (χ² = 3.62, p ≈ 0.61, df = 5) (Table 1).

**Table 1:** Clinical staging of cases (N = 62) and association with PD-L1, ALK, and ROS1 markers. ADC: adenocarcinoma, PD-L1: programmed death ligand-1, ALK: anaplastic lymphoma kinase, ROS1: c-ros oncogene 1, df: degree of freedom

Clinical stage	PD-L1 positive	Chi-square test result			ALK positive	Chi-square test result			ROS1 positive	Chi-square test result			ADC count (%)
IIA	2 (25%)	χ² = 11.12	p ≈ 0.048	df = 5	1 (12.5%)	χ² = 8.58	p ≈ 0.13	df = 5	0 (0%)	χ² = 3.62	p ≈ 0.61	df = 5	8 (12.9%)
IIIA	3 (21.4%)				0 (0%)				1 (7.1%)				14 (22.6%)
IIIB	5 (31.3%)				1 (6.3%)				0 (0%)				16 (25.8%)
IIIC	4 (100%)				1 (25%)				1 (25%)				4 (6.5%)
IVA	5 (50%)				1 (10%)				0 (0%)				10 (16.1%)
IVB	5 (50%)				0 (0%)				1 (10%)				10 (16.1%)
Total	24 (38.7%)				4 (6.5%)				3 (4.8%)				62 (100%)

Among 38 (61.2%) cases with PD-L1 < 1% or negative, only one (1.61%) was ALK-positive and none were ROS1-positive. In the 15 (24.19%) cases with PD-L1 1%-49%, there were one (1.61%) ALK-positive and two (3.23%) ROS1-positive cases. For the nine (14.52%) cases with PD-L1 > 50%, two (3.23%) were ALK-positive and one (1.61%) was ROS1-positive (Table 2).

**Table 2:** Correlation of PD-L1 TPS score with ALK and ROS1 positivity. PD-L1: programmed death ligand-1, ALK: anaplastic lymphoma kinase, ROS1: c-ros oncogene 1, TPS: Tumor Proportion Score

PD-L1 TPS	PD-L1	ALK positive	ROS1 positive
<1% or negative	38 (61.29%)	1 (1.61%)	0 (0%)
1%-49%	15 (24.19%)	1 (1.61%)	2 (3.23%)
>50%	9 (14.52%)	2 (3.23%)	1 (1.61%)

**Figure 1:**
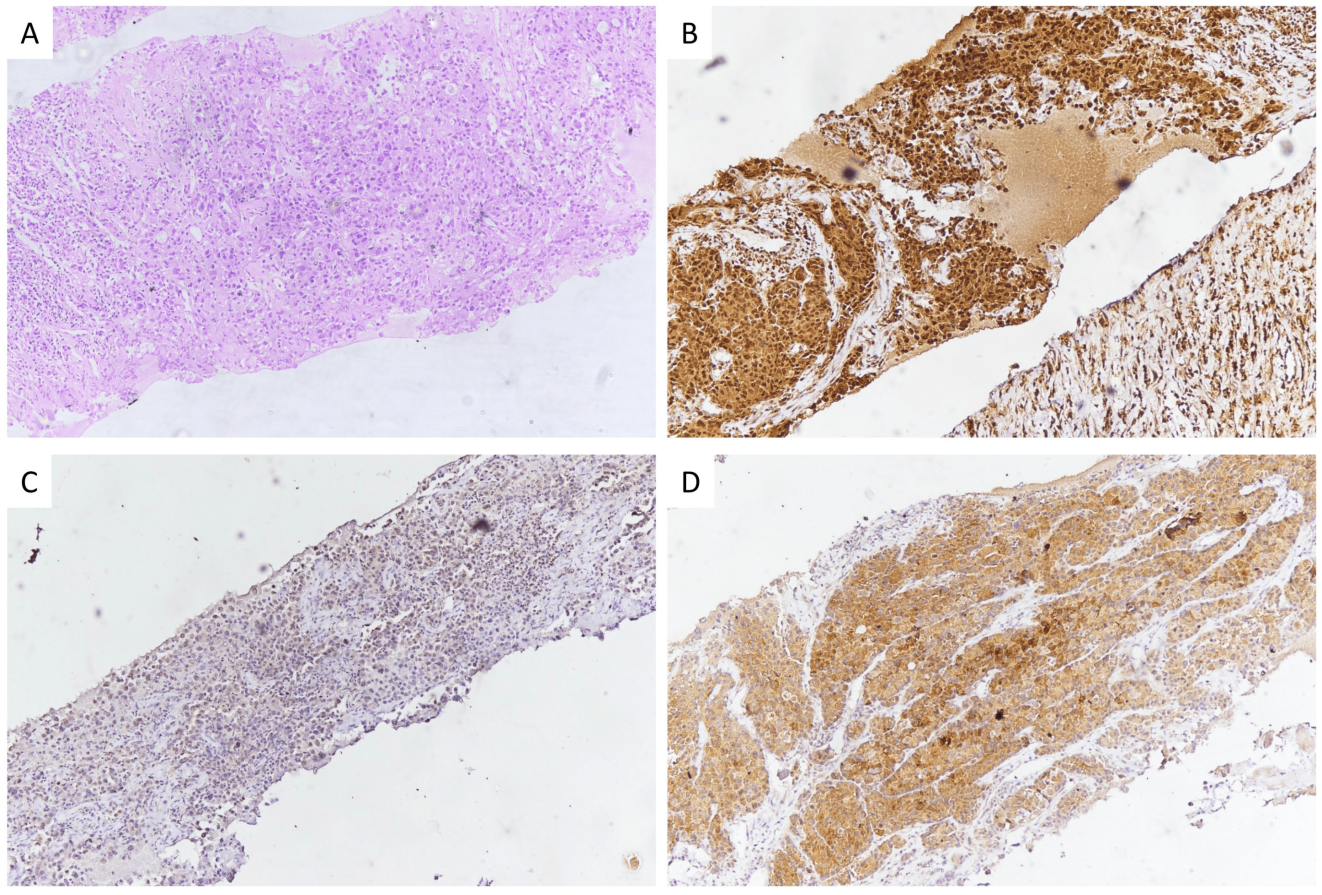
(A) Microphotograph of a Tru-cut biopsy from a lung lesion displaying infiltrating tumor cells arranged in sheets, ×400. (B) Tumor cells show PD-L1 positivity (>50%), ×400. (C) Tumor cells show ALK positivity, ×400. (D) Tumor cells show ROS1 positivity, ×400. PD-L1: programmed death ligand-1, ALK: anaplastic lymphoma kinase, ROS1: c-ros oncogene 1 Source: A case of lung carcinoma’s Tru-cut biopsy received in the Department of Pathology and Laboratory Medicine, AIIMS, Raipur, Chhattisgarh

The analysis indicates no conclusive link between PD-L1 expression and ALK or ROS1 positivity. The calculated odds ratio is about 33.1 (95% confidence interval (CI): 2.1-518.5) for ALK, with a p-value of approximately 0.058. Similarly, for ROS1, the odds ratio is around 27.1 (95% CI: 1.7-424), with a p-value near 0.058. These results suggest a trend toward association, but they do not reach statistical significance, indicating that PD-L1 positivity may not be strongly correlated with ALK or ROS1 alterations in this cohort. The majority of mutations are identified in solid subtype ADC (Figure 1).

Three cases exhibit simultaneous positivity for both ALK and ROS1, resulting in an odds ratio of roughly 1.17 (95% CI: 0.20-6.58). The p-value is approximately 0.95, which suggests there is no statistically meaningful correlation between ALK and ROS1 copositivity in this cohort.

## Discussion

In the past 10 years, molecular research on lung cancer has identified specific mutations, particularly in lung ADC, which have paved the way for targeted therapies. These treatments focus on particular mutations and have demonstrated substantial survival advantages for certain patients with advanced lung cancer [7]. In Western countries and most Asian countries, ADC has surpassed SCC as the predominant histological type of lung cancer [8]. This change in trends appears to be partially linked to evolving smoking behaviors and a rising occurrence of lung cancer among women and non-smokers.

The PD-1/PD-L1 pathway is a rapidly advancing and promising focus within tumor immunology. Tumors exploit the interaction between PD-1 and PD-L1 as a major strategy to evade immune surveillance, shifting the balance toward immune suppression and tumor growth. High levels of PD-L1 expression are associated with worse prognosis, as they inhibit T-cell activation and create a tumor microenvironment dominated by Th2 cytokines that facilitate tumor progression [9-11]. In our current study, advanced stages of ADC show a strong correlation with positive PD-L1 expression, suggesting that such patients are likely to benefit from immunotherapy. Additionally, without immunotherapy, elevated PD-L1 levels serve as a negative prognostic indicator, highlighting its role not only as a predictive biomarker but also as a marker of aggressive disease progression when appropriate targeted treatment is not administered. However, in contrast with ALK and ROS1, no significant correlation was found between PD-L1 positivity and either marker. Similar findings were reported by Onur et al. [12]. Therefore, each mutation should be evaluated independently in patients with lung adenocarcinoma. However, Wang et al. found that patients with ALK+PD-L1+ had shorter progression-free survival (PFS) and overall survival (OS) than those with ALK+PD-L1- cohort [13].

Oncogenic drivers in NSCLC, including epidermal growth factor receptor (EGFR), ALK, and ROS1 alterations, are typically mutually exclusive; there have been few reports on the concomitant existence of EGFR-ALK [14], EGFR-ROS1 [15], and ALK-ROS1 mutations [16]. However, in the present study, no correlation between ALK-ROS1 co-mutation was found. Currently, testing for BRAF, RET, ERBB2 (HER2), MET, and KRAS mutations is not recommended as standalone assessments. Instead, these are typically analyzed within broader testing panels, either at the initial stage or after negative results for EGFR, ALK, and ROS1 have been obtained [7]. Several studies have documented the prevalence of EGFR mutations in the Indian population; however, research on ROS1 and ALK mutations remains limited and less extensively studied (Table 3) [17-28].

**Table 3:** Frequency of specific mutation found in various Indian studies. EGFR: epidermal growth factor receptor, PD-L1: programmed death ligand-1, ALK: anaplastic lymphoma kinase, ROS1: c-ros oncogene 1, TMH: Tata Memorial Hospital

Study	Place of investigation	EGFR	ALK	ROS1	PD-L1
Choughule et al. (2013) [19]	India	19.5% Central India	-	-	-
		22.8% East India			
		23.4% North India			
		26.7% South India			
		27.5% West India			
Rana et al. (2018) [20]	Pune	35.5%	7.6%	-	-
Noronha et al. (2013) [21]	TMH Mumbai	36.44%	-	-	-
Kasana et al. (2016) [22]	Jammu and Kashmir	35.1%	-	-	-
Pungliya et al. (2014) [23]	Delhi	68+/-17% South India	-	-	-
		41+/- 21% North India			
Kumari et al. (2019) [24]	Lucknow	36.5%	-	-	-
Jain et al. (2019) [25]	Hyderabad	-	-	0.44%	-
Mehta et al. (2020) [26]	Delhi	35%	-	2.8%	-
Kumar et al. (2020) [27]	Delhi	10.9%	17.1%	-	33.6%
Suryavanshi et al. (2017) [28]	Delhi	-	-	4.1%	-
Joshi et al. (2019) [17]	THM Mumbai	-	-	2.9%	-
Munde et al. (2024) [18]	Mumbai	29.1%	8.1%	3.5%	-
Presented study	Raipur	-	6.45%	4.84%	39%

Literature suggests that ADC is categorized into five main subtypes, each displaying distinct features: lepidic-predominant adenocarcinoma (LPA), acinar-predominant adenocarcinoma (APA), micropapillary-predominant adenocarcinoma (MPA), papillary-predominant adenocarcinoma (PPA), and solid-predominant adenocarcinoma (SPA) [29]. LPA usually presents as low-grade cancer with a favorable prognosis, whereas APA and PPA are considered to have moderate levels of malignancy. The MPA and SPA subtypes are high-grade forms, associated with poorer clinical outcomes, with MPA notably demonstrating a significant likelihood of metastasis [29]. A few studies also found molecular and morphological correlations (Table 4) [30-32].

**Table 4:** Predominant pattern of ADC and mutations in various studies. EGFR: epidermal growth factor receptor, PD-L1: programmed death ligand-1, ALK: anaplastic lymphoma kinase, ROS1: c-ros oncogene 1

Study	EGFR	ALK	ROS1	PD-L1
Ninomiya et al. (2009) [31]	Hobnail cell morphology (lepidic)	-	-	-
Wang et al. (2014) [32]	-	Adenosquamous morphology	-	-
Sholl et al. (2013) [30]	-	-	Adenosquamous morphology with psammoma calcification	-
Present study	-	Solid	Solid	Solid

Studies have documented associations between specific mutations and each subtype: LPA with RBM10 and PIK3CA, MPA with KEAP1, APA with TP53 and EGFR, PPA with EGFR and KEAP1, STK11, and SMARCA, and SPA with TP53, STK11, and SMARCA1 [33]. In the present study, most mutations are found in the solid type of ADC. Those studies provide a foundation for the development of a future WHO classification based on histo-molecular features.

Implications for clinical practice and precision medicine

The independence of PD-L1 expression from ALK and ROS1 alterations has profound implications for clinical practice. First, it indicates that comprehensive biomarker testing must encompass all relevant markers (EGFR, ALK, ROS1, PD-L1, and potentially others) rather than relying on algorithmic testing based on the presence or absence of one marker. Patients with ALK-positive or ROS1-positive tumors cannot be presumed to have concurrent PD-L1 expression and should still undergo formal PD-L1 testing. Conversely, PD-L1-negative patients may still harbor ALK or ROS1 alterations responsive to targeted tyrosine kinase inhibitors.

The absence of significant correlation among these biomarkers supports the development of multiplex diagnostic approaches that assess multiple targets simultaneously, potentially through multiplex IHC or NGS panels. Such integrated testing strategies would provide comprehensive molecular characterization in a single workflow, reducing turnaround time and costs while ensuring that no clinically relevant alteration is overlooked.

Limitation of the study

ROS1 positivity was not evaluated using FISH or PCR; instead, PCR-positive tissue was employed for the validation of IHC results. The findings suggest a potentially strong association between PD-L1 positivity and ALK/ROS1 positivity, but due to wide confidence intervals and borderline p-values, the results are not statistically significant, indicating the need for further investigation with larger sample sizes.

## Conclusions

PD-L1 expression demonstrates a statistically significant association with advanced clinical staging in lung adenocarcinoma, establishing its role as a biomarker for disease progression. However, PD-L1 expression remains independent of both ALK and ROS1 alterations in this cohort, suggesting that these biomarkers operate through distinct molecular pathways in lung adenocarcinoma pathogenesis. Additionally, ALK and ROS1 alterations show no significant correlation with each other or with clinical stage, confirming the mutual exclusivity of these driver mutations. These findings underscore the critical importance of comprehensive, multiplex biomarker testing strategies that assess all relevant molecular alterations regardless of the status of others, ensuring optimal patient selection for targeted therapies and immunotherapy in the era of precision medicine. The predominance of these mutations in the solid adenocarcinoma subtype in our cohort merits further investigation to clarify potential geographic variations in histo-molecular correlations within the Indian population.
